# Bis(4-amino­pyridinium) dichromate(VI)

**DOI:** 10.1107/S1600536814013579

**Published:** 2014-06-14

**Authors:** Sonia Trabelsi, Thierry Roisnel, Hassouna Dhaouadi, Houda Marouani

**Affiliations:** a Laboratoire de Chimie des Matériaux, Faculté des Sciences de Bizerte, 7021 Zarzouna Bizerte, Tunisia; b Centre de Diffractométrie X, UMR 6226 CNRS, Unité Sciences Chimiques de Rennes, Université de Rennes I, 263 Avenue du Général Leclerc, 35042 Rennes, France; c Laboratoire des Matériaux Utiles, Institut National de Recherche et d’Analyse Physico-chimique, Pole Technologique de Sidi-Thabet, 2020 Tunis, Tunisia

## Abstract

The asymmetric unit of the title salt, (C_5_H_7_N_2_)_2_[Cr_2_O_7_], contains four independent cations and two independent dichromate anions. The crystal structure consists of discrete dichromate anions with an eclipsed conformation stacked in layers parallel to (010) at *y* = 1/4 and *y* = 3/4. These layers are linked *via* 4-amino­pyridinium cations by N—H⋯O and weak C—H⋯O hydrogen bonds, forming a three-dimensional supra­molecular network. In addition, π–π inter­actions are present in this structure; the shortest distance separating mean planes through 4-amino­pyridinium cations is 3.679 (6) Å.

## Related literature   

For properties of pyridine-based compounds, see: Patani & LaVoie (1996 ▶); Ma & Huang (2003 ▶). For related structures, see: Trabelsi *et al.* (2012 ▶); Lennartson & Håkansson (2009 ▶); Fun *et al.* (2009 ▶); Ramesh *et al.* (2010 ▶). For aromatic π–π stacking inter­actions, see: Janiak (2000 ▶).
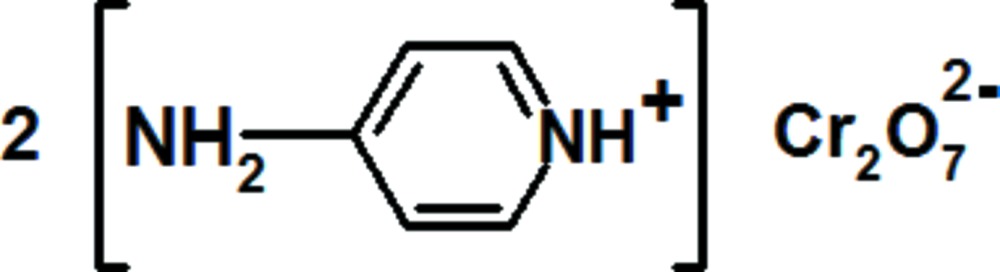


## Experimental   

### 

#### Crystal data  

(C_5_H_7_N_2_)_2_[Cr_2_O_7_]
*M* *_r_* = 406.25
Monoclinic, 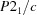

*a* = 13.8505 (4) Å
*b* = 16.2486 (4) Å
*c* = 15.2586 (4) Å
β = 118.923 (2)°
*V* = 3005.65 (14) Å^3^
*Z* = 8
Mo *K*α radiation
μ = 1.49 mm^−1^
*T* = 150 K
0.58 × 0.5 × 0.4 mm

#### Data collection  

Bruker APEXII diffractometer
Absorption correction: multi-scan (*SADABS*; Bruker, 2006 ▶) *T*_min_ = 0.469, *T*_max_ = 0.552
23461 measured reflections
6868 independent reflections
5506 reflections with *I* > 2σ(*I*)
*R*_int_ = 0.042

#### Refinement  

*R*[*F* ^2^ > 2σ(*F* ^2^)] = 0.035
*wR*(*F* ^2^) = 0.097
*S* = 1.07
6868 reflections
415 parameters
H-atom parameters constrained
Δρ_max_ = 0.41 e Å^−3^
Δρ_min_ = −0.58 e Å^−3^

### 

Data collection: *APEX2* (Bruker, 2006 ▶); cell refinement: *SAINT* (Bruker, 2006 ▶); data reduction: *SAINT*; program(s) used to solve structure: *SIR97* (Altomare *et al.*, 1999 ▶); program(s) used to refine structure: *SHELXL97* (Sheldrick, 2008 ▶); molecular graphics: *DIAMOND* (Brandenburg & Putz, 2005 ▶); software used to prepare material for publication: *WinGX* (Farrugia, 2012 ▶) and *CRYSCAL* (T. Roisnel, local program).

## Supplementary Material

Crystal structure: contains datablock(s) I, global. DOI: 10.1107/S1600536814013579/bh2500sup1.cif

Structure factors: contains datablock(s) I. DOI: 10.1107/S1600536814013579/bh2500Isup2.hkl

Supporting information file. DOI: 10.1107/S1600536814013579/bh2500Isup3.cml

CCDC reference: 1007743

Additional supporting information:  crystallographic information; 3D view; checkCIF report

## Figures and Tables

**Table 1:** Hydrogen-bond geometry (Å, °)

*D*—H⋯*A*	*D*—H	H⋯*A*	*D*⋯*A*	*D*—H⋯*A*
N1—H*N*1⋯O10^i^	0.86	2.13	2.842 (3)	140
N2—H2*A*⋯O9^ii^	0.86	2.19	3.022 (3)	162
N2—H2*B*⋯O11^iii^	0.86	2.13	2.978 (3)	167
N3—H*N*3⋯O5^iv^	0.86	2.14	2.871 (3)	142
N4—H4*A*⋯O13^iii^	0.86	2.32	3.067 (3)	145
N4—H4*B*⋯O14^ii^	0.86	2.14	2.965 (3)	160
N5—H*N*5⋯O5^ii^	0.86	2.11	2.890 (3)	151
N6—H6*A*⋯O2^iv^	0.86	2.45	2.998 (3)	122
N6—H6*B*⋯O14^iv^	0.86	2.10	2.923 (3)	160
N7—H*N*7⋯O4^v^	0.86	2.22	2.927 (2)	139
N8—H8*A*⋯O8^iv^	0.86	2.31	3.121 (3)	156
N8—H8*B*⋯O11	0.86	2.17	3.033 (3)	176
C1—H1⋯O7^vi^	0.93	2.43	3.305 (3)	156
C5—H5⋯O6^iv^	0.93	2.28	3.192 (3)	165
C6—H6⋯O1^vi^	0.93	2.31	3.204 (3)	161
C9—H9⋯O12^ii^	0.93	2.47	3.359 (3)	161
C10—H10⋯O2^iv^	0.93	2.40	3.209 (3)	145
C11—H11⋯O1^ii^	0.93	2.41	3.205 (3)	144
C14—H14⋯O12	0.93	2.46	3.248 (3)	143
C15—H15⋯O2^v^	0.93	2.32	3.244 (3)	171
C16—H16⋯O7^ii^	0.93	2.37	3.197 (3)	148

Symmetry codes: (i) 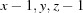; (ii) 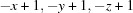; (iii) 
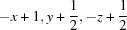
; (iv) 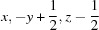; (v) 
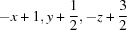
; (vi) 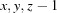.

**Fig. 1.**
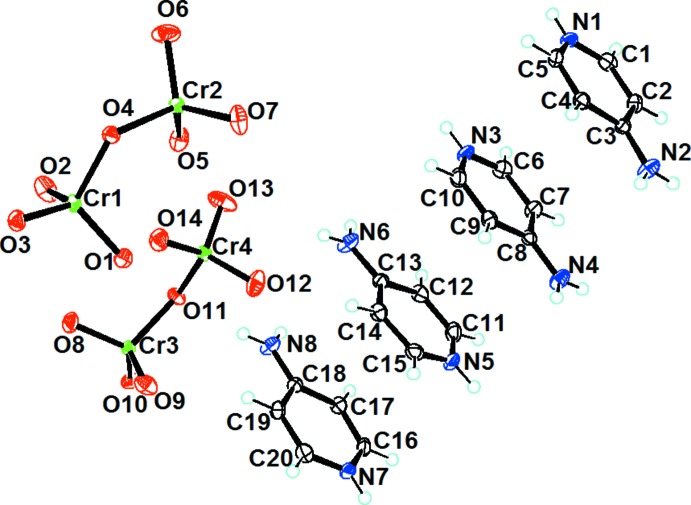
An ORTEP view of (I) with the atom-labeling scheme. Displacement ellipsoids are drawn at the 30% probability level. H atoms are represented as small spheres of arbitrary radii.

**Fig. 2.**
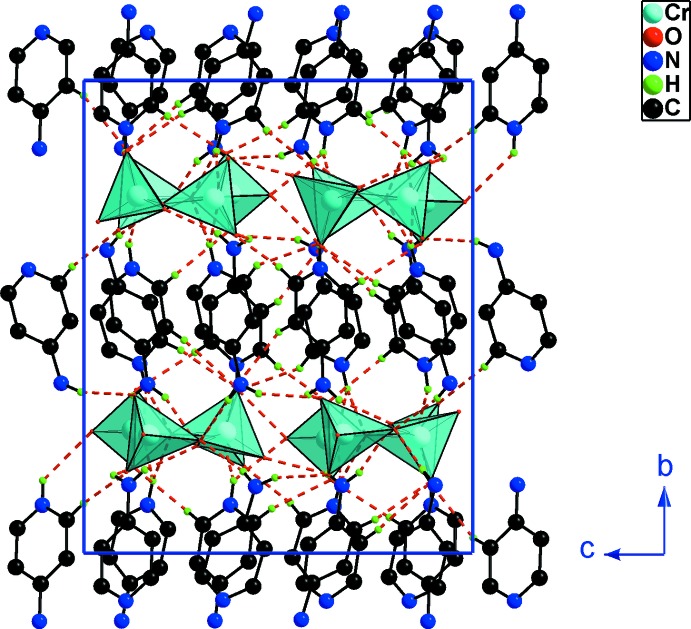
Projection of (I) along the a axis. The H-atoms not involved in H-bonding are omitted.

**Fig. 3.**
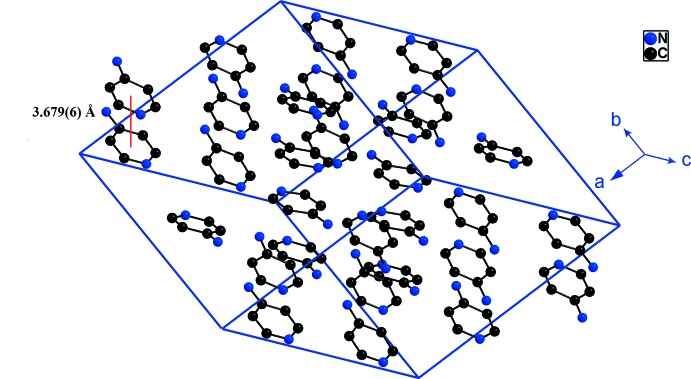
Perspective view showing the π—π interactions between adjacent 4-aminopyridinium groups. Dichromate anions have been omitted for clarity.

## supplementary crystallographic information

### S1. Comment

Usually, pyridine can serve as an efficient bioisostere of benzene in drug design, and considerable interest has been shown in pyridine derivatives in the field of modern agrochemistry and medicinal chemistry, because substitution of the benzene by pyridine may result in good biological activity and low toxicity of molecules containing the pyridyl moiety (Patani & LaVoie, 1996; Ma & Huang, 2003). In this work, we report the preparation and the structural investigation of a new organic dichromate, (C_5_H_7_N_2_)_2_·Cr_2_O_7_, (I).

The asymmetric unit of (I) is composed of two independent dichromate anions and four independent 4-aminopyridinium cations (Fig. 1). The structure of the compound consists of discrete dichromate ions with an eclipsed conformation stacked in layers parallel to the (010) plane at *y* = 0.25 and 0.75. These layers are linked *via* the 4-aminopidridinium cations by N—H···O and C—H···O hydrogen bonds, forming a three-dimensional supramolecular network as shown in figure 2.

Interatomic bond lengths and angles of the dichromate anions spread respectively within the ranges [1.6038 (17)–1.8059 (17) Å] and [105.42 (8)–112.22 (10)°] for O—Cr—O angles and [120.12 (9)–125.63 (9)°] for Cr—O—Cr angles. These geometrical features have also been noticed in other related crystal structure (Trabelsi *et al.*, 2012; Lennartson & Håkansson, 2009).

In this atomic arrangement four independent 4-aminopyridinium cations are present. Examination of the organic cations shows that the bond distances and angles show no significant difference from those obtained in oxalate and picrate salts involving the same organic group (Fun *et al.*, 2009; Ramesh *et al.*, 2010). Each 4-aminopyridinium cations is planar, with a maximum deviation of 0.0024 (2) Å. The shortest inter-planar distance between nearby pyridine rings is 3.679 (6) Å, which is much shorter than 3.80 Å, indicating the formation of π–π interactions (Fig. 3, Janiak, 2000).

The established H-bonds of types N—H···O and C—H···O involve O atoms of the dichromate anions as acceptors, and the protonated N atoms and carbon atoms of 4-aminopyridinium as donors.

### S2. Experimental

Single crystals of the title compound were prepared at room temperature by dissolving CrO_3_ (0.10 g, 1 mmol) and 4-aminopyridine (Fampridine, 0.09 g, 1 mmol) in distilled water (20 ml). The resulting solution was stirred during 1 h, filtered and then evaporated slowly at room temperature until the formation of orange prismatic single crystals.

### S3. Refinement

All H atoms were located in a difference map. Nevertheless, they were geometrically placed and refined using a riding model, with C—H = 0.93 Å, N—H = 0.86 Å, and with *U*_iso_(H) = 1.2*U*_eq_(carrier C or N).

### Crystal data

**Table:**

(C_5_H_7_N_2_)_2_[Cr_2_O_7_]	*F*(000) = 1648
*M**_r_* = 406.25	*D*_x_ = 1.796 Mg m^−^^3^
Monoclinic, *P*2_1_/*c*	Mo *K*α radiation, λ = 0.71073 Å
Hall symbol: -P 2ybc	Cell parameters from 5472 reflections
*a* = 13.8505 (4) Å	θ = 2.7–27.3°
*b* = 16.2486 (4) Å	µ = 1.49 mm^−^^1^
*c* = 15.2586 (4) Å	*T* = 150 K
β = 118.923 (2)°	Prism, orange
*V* = 3005.65 (14) Å^3^	0.58 × 0.5 × 0.4 mm
*Z* = 8	

### Data collection

**Table:**

Bruker APEXII diffractometer	5506 reflections with *I* > 2σ(*I*)
Graphite monochromator	*R*_int_ = 0.042
CCD rotation images, thin slices scans	θ_max_ = 27.5°, θ_min_ = 2.9°
Absorption correction: multi-scan (*SADABS*; Bruker, 2006)	*h* = −17→17
*T*_min_ = 0.469, *T*_max_ = 0.552	*k* = −16→21
23461 measured reflections	*l* = −17→19
6868 independent reflections	

### Refinement

**Table:**

Refinement on *F*^2^	Primary atom site location: structure-invariant direct methods
Least-squares matrix: full	Secondary atom site location: difference Fourier map
*R*[*F*^2^ > 2σ(*F*^2^)] = 0.035	Hydrogen site location: inferred from neighbouring sites
*wR*(*F*^2^) = 0.097	H-atom parameters constrained
*S* = 1.07	*w* = 1/[σ^2^(*F*_o_^2^) + (0.0409*P*)^2^ + 1.1738*P*] where *P* = (*F*_o_^2^ + 2*F*_c_^2^)/3
6868 reflections	(Δ/σ)_max_ = 0.001
415 parameters	Δρ_max_ = 0.41 e Å^−^^3^
0 restraints	Δρ_min_ = −0.58 e Å^−^^3^
0 constraints	

### Fractional atomic coordinates and isotropic or equivalent isotropic displacement parameters (Å^2^)

**Table:**

	*x*	*y*	*z*	*U*_iso_*/*U*_eq_	
Cr1	0.30978 (3)	0.25913 (2)	0.87600 (3)	0.01226 (10)	
Cr2	0.14056 (3)	0.25667 (2)	0.64724 (3)	0.01334 (10)	
O1	0.36183 (14)	0.34529 (10)	0.86547 (12)	0.0217 (4)	
O2	0.37986 (14)	0.18108 (11)	0.87204 (13)	0.0251 (4)	
O3	0.30456 (14)	0.25734 (9)	0.97973 (12)	0.0175 (4)	
O4	0.16957 (13)	0.25140 (9)	0.77567 (12)	0.0141 (3)	
O5	0.25558 (15)	0.24640 (9)	0.64455 (13)	0.0206 (4)	
O6	0.05649 (17)	0.18307 (12)	0.58819 (13)	0.0335 (5)	
O7	0.08641 (15)	0.34537 (11)	0.60243 (13)	0.0257 (4)	
Cr3	0.86884 (3)	0.24904 (2)	0.83351 (3)	0.01316 (10)	
Cr4	0.63121 (3)	0.25479 (2)	0.63638 (3)	0.01368 (10)	
O8	0.85329 (15)	0.17165 (11)	0.89157 (13)	0.0285 (4)	
O9	0.84062 (15)	0.33450 (11)	0.87063 (13)	0.0256 (4)	
O10	0.99257 (14)	0.25074 (9)	0.84773 (13)	0.0187 (4)	
O11	0.77849 (14)	0.23816 (9)	0.70058 (12)	0.0179 (4)	
O12	0.60940 (16)	0.35133 (11)	0.61015 (15)	0.0314 (5)	
O13	0.58360 (15)	0.20101 (13)	0.53587 (14)	0.0334 (5)	
O14	0.57985 (15)	0.22726 (11)	0.70728 (13)	0.0261 (4)	
N1	0.10279 (16)	0.40072 (12)	−0.14295 (15)	0.0211 (5)	
HN1	0.0888	0.3498	−0.1597	0.025*	
C1	0.1345 (2)	0.45034 (15)	−0.19490 (19)	0.0210 (5)	
H1	0.1399	0.4292	−0.2490	0.025*	
C2	0.1588 (2)	0.53063 (14)	−0.16971 (18)	0.0179 (5)	
H2	0.1804	0.5642	−0.2065	0.021*	
C3	0.15119 (19)	0.56323 (14)	−0.08699 (17)	0.0156 (5)	
C4	0.11508 (19)	0.50918 (14)	−0.03606 (18)	0.0181 (5)	
H4	0.1068	0.5284	0.0174	0.022*	
C5	0.0925 (2)	0.42948 (15)	−0.06501 (19)	0.0211 (5)	
H5	0.0696	0.3942	−0.0307	0.025*	
N2	0.17652 (18)	0.64076 (12)	−0.05892 (16)	0.0237 (5)	
H2A	0.1712	0.6594	−0.0087	0.028*	
H2B	0.1983	0.6726	−0.0909	0.028*	
N3	0.36147 (17)	0.39842 (12)	0.12358 (15)	0.0212 (5)	
HN3	0.3460	0.3468	0.1153	0.025*	
C6	0.3965 (2)	0.43597 (15)	0.06512 (19)	0.0217 (5)	
H6	0.4039	0.4059	0.0168	0.026*	
C7	0.4210 (2)	0.51765 (14)	0.07674 (18)	0.0188 (5)	
H7	0.4446	0.5433	0.0360	0.023*	
C8	0.41064 (18)	0.56376 (14)	0.15032 (17)	0.0154 (5)	
C9	0.37277 (19)	0.52161 (15)	0.20893 (17)	0.0176 (5)	
H9	0.3633	0.5497	0.2573	0.021*	
C10	0.3502 (2)	0.43966 (15)	0.19426 (18)	0.0213 (5)	
H10	0.3267	0.4119	0.2338	0.026*	
N4	0.43612 (18)	0.64317 (12)	0.16345 (16)	0.0229 (5)	
H4A	0.4593	0.6673	0.1269	0.027*	
H4B	0.4295	0.6705	0.2085	0.027*	
N5	0.66256 (17)	0.60129 (12)	0.39258 (16)	0.0238 (5)	
HN5	0.6758	0.6531	0.3940	0.029*	
C11	0.6760 (2)	0.55244 (16)	0.32819 (19)	0.0244 (6)	
H11	0.6991	0.5753	0.2856	0.029*	
C12	0.6565 (2)	0.47016 (15)	0.32428 (18)	0.0190 (5)	
H12	0.6668	0.4372	0.2797	0.023*	
C13	0.62036 (19)	0.43485 (14)	0.38822 (18)	0.0165 (5)	
C14	0.6076 (2)	0.48867 (14)	0.45475 (18)	0.0186 (5)	
H14	0.5847	0.4680	0.4986	0.022*	
C15	0.6286 (2)	0.57029 (15)	0.45493 (18)	0.0208 (5)	
H15	0.6194	0.6052	0.4986	0.025*	
N6	0.59854 (18)	0.35547 (12)	0.38491 (17)	0.0243 (5)	
H6A	0.5759	0.3352	0.4239	0.029*	
H6B	0.6069	0.3240	0.3438	0.029*	
N7	0.89889 (16)	0.60146 (12)	0.66303 (15)	0.0190 (4)	
HN7	0.9067	0.6532	0.6768	0.023*	
C16	0.9173 (2)	0.57268 (15)	0.58964 (18)	0.0193 (5)	
H16	0.9388	0.6088	0.5549	0.023*	
C17	0.9048 (2)	0.49117 (14)	0.56583 (18)	0.0176 (5)	
H17	0.9179	0.4719	0.5152	0.021*	
C18	0.87181 (18)	0.43586 (14)	0.61778 (17)	0.0147 (5)	
C19	0.8537 (2)	0.46897 (14)	0.69469 (17)	0.0173 (5)	
H19	0.8317	0.4348	0.7307	0.021*	
C20	0.8684 (2)	0.55068 (14)	0.71542 (18)	0.0193 (5)	
H20	0.8574	0.5720	0.7664	0.023*	
N8	0.85990 (17)	0.35635 (12)	0.59675 (16)	0.0224 (5)	
H8A	0.8723	0.3373	0.5505	0.027*	
H8B	0.8398	0.3237	0.6293	0.027*	

### Atomic displacement parameters (Å^2^)

**Table:**

	*U*^11^	*U*^22^	*U*^33^	*U*^12^	*U*^13^	*U*^23^
Cr1	0.01379 (19)	0.01437 (19)	0.00998 (19)	0.00100 (14)	0.00682 (15)	0.00061 (13)
Cr2	0.0164 (2)	0.0147 (2)	0.01068 (19)	−0.00082 (14)	0.00786 (16)	−0.00082 (13)
O1	0.0222 (9)	0.0246 (9)	0.0173 (9)	−0.0072 (7)	0.0087 (8)	0.0028 (7)
O2	0.0254 (10)	0.0302 (10)	0.0190 (9)	0.0122 (8)	0.0101 (8)	−0.0010 (7)
O3	0.0242 (9)	0.0174 (9)	0.0133 (8)	−0.0004 (7)	0.0110 (7)	0.0004 (6)
O4	0.0140 (8)	0.0178 (9)	0.0118 (8)	−0.0018 (6)	0.0074 (7)	−0.0005 (6)
O5	0.0264 (10)	0.0203 (9)	0.0237 (10)	0.0048 (7)	0.0189 (8)	0.0033 (7)
O6	0.0422 (12)	0.0414 (12)	0.0202 (10)	−0.0249 (10)	0.0176 (9)	−0.0157 (8)
O7	0.0315 (11)	0.0282 (10)	0.0198 (9)	0.0136 (8)	0.0143 (8)	0.0090 (7)
Cr3	0.0135 (2)	0.0144 (2)	0.01207 (19)	−0.00019 (14)	0.00655 (16)	0.00043 (14)
Cr4	0.0156 (2)	0.0147 (2)	0.01179 (19)	0.00095 (14)	0.00741 (16)	−0.00091 (13)
O8	0.0246 (10)	0.0328 (11)	0.0250 (10)	−0.0072 (8)	0.0095 (8)	0.0110 (8)
O9	0.0253 (10)	0.0289 (10)	0.0210 (9)	0.0056 (8)	0.0101 (8)	−0.0072 (8)
O10	0.0162 (9)	0.0168 (9)	0.0233 (9)	−0.0012 (6)	0.0097 (8)	−0.0010 (7)
O11	0.0169 (9)	0.0228 (9)	0.0128 (8)	0.0018 (7)	0.0063 (7)	−0.0024 (7)
O12	0.0348 (12)	0.0212 (10)	0.0418 (12)	0.0093 (8)	0.0213 (10)	0.0123 (8)
O13	0.0224 (10)	0.0478 (13)	0.0247 (10)	0.0023 (9)	0.0072 (8)	−0.0187 (9)
O14	0.0280 (10)	0.0314 (10)	0.0237 (10)	−0.0016 (8)	0.0164 (9)	0.0043 (8)
N1	0.0185 (11)	0.0118 (10)	0.0255 (12)	−0.0007 (8)	0.0046 (9)	−0.0024 (8)
C1	0.0188 (13)	0.0218 (13)	0.0200 (13)	0.0039 (10)	0.0074 (11)	−0.0039 (10)
C2	0.0185 (13)	0.0184 (12)	0.0194 (13)	0.0039 (10)	0.0113 (11)	0.0036 (10)
C3	0.0121 (11)	0.0141 (11)	0.0191 (12)	0.0003 (9)	0.0065 (10)	0.0010 (9)
C4	0.0164 (12)	0.0219 (13)	0.0159 (12)	−0.0011 (10)	0.0078 (10)	0.0021 (9)
C5	0.0187 (13)	0.0189 (12)	0.0223 (13)	−0.0001 (10)	0.0072 (11)	0.0071 (10)
N2	0.0328 (13)	0.0157 (10)	0.0320 (13)	−0.0039 (9)	0.0233 (11)	−0.0041 (9)
N3	0.0219 (11)	0.0107 (10)	0.0277 (12)	−0.0011 (8)	0.0094 (10)	0.0004 (8)
C6	0.0221 (13)	0.0216 (13)	0.0225 (13)	0.0008 (10)	0.0117 (11)	−0.0039 (10)
C7	0.0244 (14)	0.0179 (12)	0.0187 (13)	−0.0006 (10)	0.0140 (11)	−0.0008 (10)
C8	0.0120 (11)	0.0162 (12)	0.0153 (12)	0.0013 (9)	0.0046 (10)	0.0000 (9)
C9	0.0173 (12)	0.0219 (12)	0.0147 (12)	0.0024 (10)	0.0085 (10)	0.0020 (9)
C10	0.0174 (13)	0.0237 (13)	0.0215 (13)	0.0008 (10)	0.0085 (11)	0.0074 (10)
N4	0.0346 (13)	0.0152 (10)	0.0272 (12)	−0.0019 (9)	0.0216 (11)	−0.0025 (9)
N5	0.0226 (12)	0.0129 (10)	0.0287 (12)	−0.0010 (9)	0.0066 (10)	0.0048 (9)
C11	0.0215 (14)	0.0286 (14)	0.0228 (14)	−0.0014 (11)	0.0105 (11)	0.0095 (11)
C12	0.0176 (13)	0.0256 (13)	0.0163 (12)	−0.0012 (10)	0.0101 (11)	−0.0011 (10)
C13	0.0137 (12)	0.0165 (12)	0.0178 (12)	−0.0004 (9)	0.0064 (10)	0.0001 (9)
C14	0.0194 (13)	0.0211 (13)	0.0171 (12)	0.0007 (10)	0.0102 (11)	0.0007 (10)
C15	0.0194 (13)	0.0176 (12)	0.0189 (13)	0.0025 (10)	0.0042 (11)	−0.0030 (10)
N6	0.0342 (13)	0.0172 (11)	0.0322 (12)	−0.0065 (9)	0.0246 (11)	−0.0050 (9)
N7	0.0203 (11)	0.0118 (10)	0.0214 (11)	0.0017 (8)	0.0072 (9)	0.0006 (8)
C16	0.0175 (13)	0.0201 (13)	0.0187 (13)	0.0008 (10)	0.0076 (10)	0.0074 (10)
C17	0.0169 (12)	0.0209 (13)	0.0173 (12)	−0.0001 (10)	0.0102 (10)	0.0009 (9)
C18	0.0114 (11)	0.0152 (11)	0.0173 (12)	0.0004 (9)	0.0067 (10)	0.0010 (9)
C19	0.0192 (13)	0.0187 (12)	0.0165 (12)	0.0018 (10)	0.0106 (10)	0.0028 (9)
C20	0.0212 (13)	0.0206 (12)	0.0169 (12)	0.0048 (10)	0.0100 (11)	−0.0010 (10)
N8	0.0312 (13)	0.0165 (10)	0.0284 (12)	−0.0045 (9)	0.0215 (11)	−0.0037 (9)

### Geometric parameters (Å, º)

**Table:**

Cr1—O2	1.6158 (17)	C7—H7	0.9300
Cr1—O1	1.6178 (17)	C8—N4	1.327 (3)
Cr1—O3	1.6193 (16)	C8—C9	1.413 (3)
Cr1—O4	1.8041 (17)	C9—C10	1.361 (3)
Cr2—O6	1.6072 (18)	C9—H9	0.9300
Cr2—O7	1.6160 (17)	C10—H10	0.9300
Cr2—O5	1.6219 (18)	N4—H4A	0.8600
Cr2—O4	1.8020 (16)	N4—H4B	0.8600
Cr3—O8	1.6115 (17)	N5—C11	1.344 (3)
Cr3—O9	1.6175 (17)	N5—C15	1.347 (3)
Cr3—O10	1.6202 (17)	N5—HN5	0.8600
Cr3—O11	1.8044 (17)	C11—C12	1.360 (3)
Cr4—O13	1.6038 (17)	C11—H11	0.9300
Cr4—O12	1.6109 (18)	C12—C13	1.415 (3)
Cr4—O14	1.6188 (17)	C12—H12	0.9300
Cr4—O11	1.8059 (17)	C13—N6	1.320 (3)
N1—C1	1.345 (3)	C13—C14	1.414 (3)
N1—C5	1.348 (3)	C14—C15	1.357 (3)
N1—HN1	0.8600	C14—H14	0.9300
C1—C2	1.356 (3)	C15—H15	0.9300
C1—H1	0.9300	N6—H6A	0.8600
C2—C3	1.419 (3)	N6—H6B	0.8600
C2—H2	0.9300	N7—C16	1.347 (3)
C3—N2	1.322 (3)	N7—C20	1.351 (3)
C3—C4	1.415 (3)	N7—HN7	0.8600
C4—C5	1.355 (3)	C16—C17	1.362 (3)
C4—H4	0.9300	C16—H16	0.9300
C5—H5	0.9300	C17—C18	1.412 (3)
N2—H2A	0.8600	C17—H17	0.9300
N2—H2B	0.8600	C18—N8	1.322 (3)
N3—C10	1.341 (3)	C18—C19	1.419 (3)
N3—C6	1.350 (3)	C19—C20	1.357 (3)
N3—HN3	0.8600	C19—H19	0.9300
C6—C7	1.360 (3)	C20—H20	0.9300
C6—H6	0.9300	N8—H8A	0.8600
C7—C8	1.413 (3)	N8—H8B	0.8600
			
O2—Cr1—O1	111.77 (10)	C8—C7—H7	119.8
O2—Cr1—O3	109.52 (8)	N4—C8—C7	120.9 (2)
O1—Cr1—O3	110.20 (8)	N4—C8—C9	121.9 (2)
O2—Cr1—O4	109.46 (8)	C7—C8—C9	117.2 (2)
O1—Cr1—O4	108.93 (8)	C10—C9—C8	119.7 (2)
O3—Cr1—O4	106.84 (8)	C10—C9—H9	120.1
O6—Cr2—O7	111.36 (10)	C8—C9—H9	120.1
O6—Cr2—O5	111.31 (9)	N3—C10—C9	121.0 (2)
O7—Cr2—O5	109.97 (9)	N3—C10—H10	119.5
O6—Cr2—O4	107.12 (8)	C9—C10—H10	119.5
O7—Cr2—O4	108.44 (8)	C8—N4—H4A	120.0
O5—Cr2—O4	108.51 (8)	C8—N4—H4B	120.0
O8—Cr3—O9	110.98 (11)	H4A—N4—H4B	120.0
O8—Cr3—O10	110.71 (9)	C11—N5—C15	121.0 (2)
O9—Cr3—O10	110.95 (9)	C11—N5—HN5	119.5
O8—Cr3—O11	109.70 (9)	C15—N5—HN5	119.5
O9—Cr3—O11	108.91 (8)	N5—C11—C12	121.2 (2)
O10—Cr3—O11	105.42 (8)	N5—C11—H11	119.4
O13—Cr4—O12	110.61 (10)	C12—C11—H11	119.4
O13—Cr4—O14	112.22 (10)	C11—C12—C13	119.8 (2)
O12—Cr4—O14	110.18 (9)	C11—C12—H12	120.1
O13—Cr4—O11	105.75 (8)	C13—C12—H12	120.1
O12—Cr4—O11	107.90 (9)	N6—C13—C14	121.7 (2)
O14—Cr4—O11	110.01 (9)	N6—C13—C12	121.3 (2)
Cr2—O4—Cr1	120.12 (9)	C14—C13—C12	116.9 (2)
Cr3—O11—Cr4	125.63 (9)	C15—C14—C13	120.3 (2)
C1—N1—C5	121.1 (2)	C15—C14—H14	119.8
C1—N1—HN1	119.5	C13—C14—H14	119.8
C5—N1—HN1	119.5	N5—C15—C14	120.8 (2)
N1—C1—C2	121.1 (2)	N5—C15—H15	119.6
N1—C1—H1	119.4	C14—C15—H15	119.6
C2—C1—H1	119.4	C13—N6—H6A	120.0
C1—C2—C3	119.8 (2)	C13—N6—H6B	120.0
C1—C2—H2	120.1	H6A—N6—H6B	120.0
C3—C2—H2	120.1	C16—N7—C20	121.3 (2)
N2—C3—C4	121.5 (2)	C16—N7—HN7	119.4
N2—C3—C2	121.4 (2)	C20—N7—HN7	119.4
C4—C3—C2	117.1 (2)	N7—C16—C17	120.5 (2)
C5—C4—C3	120.1 (2)	N7—C16—H16	119.7
C5—C4—H4	120.0	C17—C16—H16	119.7
C3—C4—H4	120.0	C16—C17—C18	120.2 (2)
N1—C5—C4	120.9 (2)	C16—C17—H17	119.9
N1—C5—H5	119.6	C18—C17—H17	119.9
C4—C5—H5	119.6	N8—C18—C17	121.5 (2)
C3—N2—H2A	120.0	N8—C18—C19	121.3 (2)
C3—N2—H2B	120.0	C17—C18—C19	117.2 (2)
H2A—N2—H2B	120.0	C20—C19—C18	119.8 (2)
C10—N3—C6	121.6 (2)	C20—C19—H19	120.1
C10—N3—HN3	119.2	C18—C19—H19	120.1
C6—N3—HN3	119.2	N7—C20—C19	121.0 (2)
N3—C6—C7	120.1 (2)	N7—C20—H20	119.5
N3—C6—H6	119.9	C19—C20—H20	119.5
C7—C6—H6	119.9	C18—N8—H8A	120.0
C6—C7—C8	120.4 (2)	C18—N8—H8B	120.0
C6—C7—H7	119.8	H8A—N8—H8B	120.0

### Hydrogen-bond geometry (Å, º)

**Table:**

*D*—H···*A*	*D*—H	H···*A*	*D*···*A*	*D*—H···*A*
N1—H*N*1···O10^i^	0.86	2.13	2.842 (3)	140
N1—H*N*1···O4^ii^	0.86	2.42	3.065 (2)	133
N2—H2*A*···O9^iii^	0.86	2.19	3.022 (3)	162
N2—H2*B*···O11^iv^	0.86	2.13	2.978 (3)	167
N3—H*N*3···O5^v^	0.86	2.14	2.871 (3)	142
N3—H*N*3···O3^ii^	0.86	2.36	3.003 (3)	132
N4—H4*A*···O13^iv^	0.86	2.32	3.067 (3)	145
N4—H4*A*···O1^iii^	0.86	2.43	3.042 (3)	128
N4—H4*B*···O14^iii^	0.86	2.14	2.965 (3)	160
N5—H*N*5···O5^iii^	0.86	2.11	2.890 (3)	151
N5—H*N*5···O3^vi^	0.86	2.48	3.094 (3)	129
N6—H6*A*···O2^v^	0.86	2.45	2.998 (3)	122
N6—H6*B*···O14^v^	0.86	2.10	2.923 (3)	160
N7—H*N*7···O10^vii^	0.86	2.26	2.900 (3)	131
N7—H*N*7···O4^vi^	0.86	2.22	2.927 (2)	139
N8—H8*A*···O8^v^	0.86	2.31	3.121 (3)	156
N8—H8*B*···O11	0.86	2.17	3.033 (3)	176
C1—H1···O7^ii^	0.93	2.43	3.305 (3)	156
C5—H5···O6^v^	0.93	2.28	3.192 (3)	165
C6—H6···O1^ii^	0.93	2.31	3.204 (3)	161
C9—H9···O12^iii^	0.93	2.47	3.359 (3)	161
C10—H10···O2^v^	0.93	2.40	3.209 (3)	145
C11—H11···O1^iii^	0.93	2.41	3.205 (3)	144
C14—H14···O12	0.93	2.46	3.248 (3)	143
C15—H15···O2^vi^	0.93	2.32	3.244 (3)	171
C16—H16···O7^iii^	0.93	2.37	3.197 (3)	148

Symmetry codes: (i) *x*−1, *y*, *z*−1; (ii) *x*, *y*, *z*−1; (iii) −*x*+1, −*y*+1, −*z*+1; (iv) −*x*+1, *y*+1/2, −*z*+1/2; (v) *x*, −*y*+1/2, *z*−1/2; (vi) −*x*+1, *y*+1/2, −*z*+3/2; (vii) −*x*+2, *y*+1/2, −*z*+3/2.
